# Potential Mechanism of Action of *meso*-Dihydroguaiaretic Acid on *Mycobacterium tuberculosis* H37Rv

**DOI:** 10.3390/molecules191220170

**Published:** 2014-12-02

**Authors:** Aldo F. Clemente-Soto, Isaías Balderas-Rentería, Gildardo Rivera, Aldo Segura-Cabrera, Elvira Garza-González, María del Rayo Camacho-Corona

**Affiliations:** 1 Facultad de Ciencias Químicas, Universidad Autónoma de Nuevo León, Av. Universidad s/n, Ciudad Universitaria, San Nicolás de los Garza, Nuevo León 66451, Mexico; E-Mails: aldo.clemente@alumnos.uaem.mx (A.F.C.-S.); isaias.balderasrn@uanl.edu.mx (I.B.-R.); 2 Centro de Biotecnología Genómica, Instituto Politécnico Nacional, Boulevard del Maestro s/n, Col. Narciso Mendoza, Reynosa, Tamaulipas 88710, Mexico; E-Mails: giriveras@ipn.mx (G.R.); aldo.segura-cabrera@cchmc.org (A.S.-C.); 3 Servicio de Gastroenterología y Departamento de Patología Clínica, Hospital, Universitario Dr. José Eleuterio González, Universidad Autónoma de Nuevo León, Madero y Aguirre Pequeño, Mitras Centro, Monterrey, Nuevo León 64460, Mexico; E-Mail: elvira_garza_gzz@yahoo.com

**Keywords:** *Mycobacterium tuberculosis* H37Rv, *meso***-**dihydroguaiaretic acid, natural product, mode of action

## Abstract

The isolation and characterization of the lignan *meso*-dihydroguaiaretic acid (MDGA) from *Larrea tridentata* and its activity against *Mycobacterial tuberculosis* has been demonstrated, but no information regarding its mechanism of action has been documented. Therefore, in this study we carry out the gene expression from total RNA obtained from *M. tuberculosis* H37Rv treated with MDGA using microarray technology, which was validated by quantitative real time polymerase chain reaction. Results showed that the alpha subunit of coenzyme A transferase of *M. tuberculosis* H37Rv is present in both geraniol and 1-and 2-methylnaphthalene degradation pathways, which are targeted by MDGA. This assumption was supported by molecular docking which showed stable interaction between MDGA with the active site of the enzyme. We propose that inhibition of coenzyme A transferase of *M. tuberculosis* H37Rv results in the accumulation of geraniol and 1-and 2-methylnaphtalene inside bacteria, causing membrane destabilization and death of the pathogen. The natural product MDGA is thus an attractive template to develop new anti-tuberculosis drugs, because its target is different from those of known anti-tubercular agents.

## 1. Introduction

Tuberculosis (TB) is the second leading cause of death from an infectious disease worldwide. Currently it is estimated that one-third of the world population is infected with *Mycobacterium tuberculosis*. There were 8.6 million of new TB cases in 2012 and 1.3 million TB deaths [1]. In addition, the presence of multi-drug resistant-TB (MDR-TB), extremely-drug resistant-TB (XDR-TB) and totally drug-resistance-TB (TDR-TB) [2] make for an urgent need to discover new drugs. These drugs must be capable of being administered to TB-HIV patients, who receive combined drug therapies which is a complex situation due to potential drug-drug interactions reducing drug levels and shared drug toxicities which may exacerbate in immunosuppression and promote adverse reactions. Furthermore, new TB drugs should be active against drug-resistant forms of *M. tuberculosis*, and act upon different molecular targets [3].

**Figure 1:**
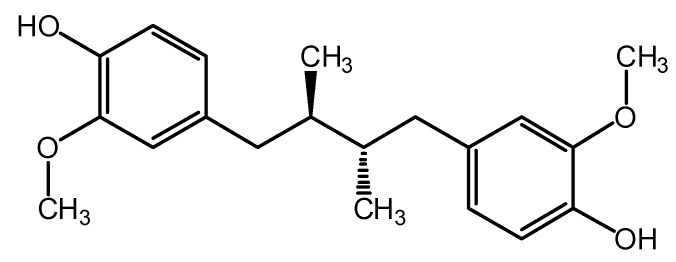
Lignan *meso*-dihydroguaiaretic acid with antimycobacterial activity.

Natural products play an important role in the discovery of new drugs; approximately 50% of drugs prescribed by physicians come from natural resources [4]. Natural products and some of their derivatives have been reported to exhibit remarkable growth inhibitory activity towards *M. tuberculosis* and some of them have been selected as prototype molecules for the development of new anti-tubercular agents [5]. In previous studies our research group isolated from *Larrea tridentata* (Zygophyllaceae) and characterized the lignan *meso*-dihydroguaiaretic acid (MDGA, Figure 1) as the anti-tubercular active compound [6] which had a minimum inhibitory concentration (MIC) in the range of 12.5 to 50 µg/mL against three MDR clinical isolates of *M. tuberculosis*, and 50 µg/mL against *M. tuberculosis* H37Rv [7].

Several lignans with antimycobacterial activity have been found [8,9,10], however very little is known about their mode of action. In this sense we found in the literature that antimycobacterial lignan ethoxycubebin obtained from *Virola flexuosa* (Myristicaceae) inhibited mycolic acid biosynthesis on bacteria [9]. During the past two decades with the advent of microarray technology, the discipline of pharmacogenomics has focused this knowledge in drug discovery and prediction of the mode of action of drugs due to the analysis of whole-genome of one organism [11]. Currently, the mode of action of lignans on *M. tuberculosis* determined using microarray analysis has not been published, therefore the aim of this study was to contribute to the knowledge of the mode of action of the lignan MDGA on *M. tuberculosis* H37Rv using microarray analysis in order to discover new targets which might be used in the design of new anti-TB drugs.

## 2. Results and Discussion

### 2.1. Growth Curve of M. tuberculosis H37Rv with Different Concentrations of MDGA

**Figure 2:**
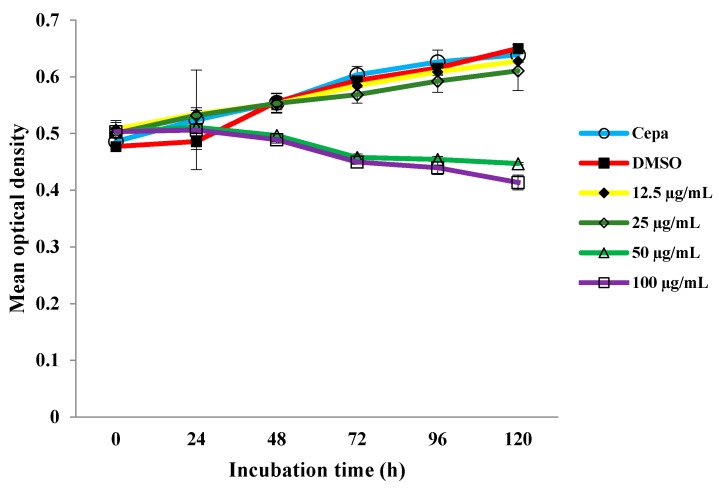
.*M. tuberculosis* H37Rv exposed to different concentrations of MDGA.

First of all we exposed bacteria to different concentrations of MDGA during 5 days. The results in Figure 2 show that 50 µg/mL of MDGA was the MIC value of MDGA which inhibited the growth of *M. tuberculosis* H37Rv after 48 h of treatment. Therefore, *M. tuberculosis* H37Rv was exposed to 50 µg/mL during 24 h, in order to promote an alteration in the gene expression of the bacteria.

### 2.2. Microarray Assay and RT-PCR

Two cultures of *M. tuberculosis* H37Rv were prepared, one culture was treated with 50 µg/mL of MDGA and the other culture received DMSO (<0.05% v/v), both cultures were incubated for 24 h. Then RNA was obtained from each bacterial culture; the synthesis and labeling of cDNA was carried out in each RNA obtained. Hybridization was done on a *M. tuberculosis* H37Rv DNA microarray chip. The chip was read on a microarray scanner, statistical analysis of gene expression was done using genArise resulting in 89 genes up-regulated (Zscore +1.5) and 17 genes down-regulated (Zscore −1.5). Classification of whole gene expression according with their functional category is shown in Table 1. Among up-regulated genes, the cell wall and cell processes (22.47%) and conserved hypotheticals (25.84%) categories are the highest genes expressed. While the down-regulated genes were related to intermediary metabolism and respiration (35.29%) as well as cell wall and cell processes (29.41%) categories in *M. tuberculosis* H37Rv.

**Table 1:** Functional category of up- and down-regulated genes observed in microarrays analysis obtained from *M. tuberculosis*H37Rv exposed to different concentrations of MDGA.

Functional Category	Up-Regulated Genes	Down-Regulated Genes
PE/PPE	11.23%	---
Conserved hypotheticals	25.84%	17.64%
Cell wall and cell processes	22.47%	29.41%
Intermediary metabolism and respiration	17.99%	35.29%
Regulatory proteins	3.37%	11.76%
Virulence, detoxification y adaptation	6.74%	5.88%
Information pathways	6.74%	---
Lipids metabolism	4.49%	---
Insertion sequences and phages	1.12%	---

Gene expression given by the microarray assay was validated by real time reverse transcription polymerase chain reaction (RT-PCR, Table 2). The results showed that microarray gene expression pattern was in agreement with the RT-PCR results. However, some genes (Rv3903c and Rv0584), showed six times greater expression in RT-PCR than the microarray. These differences have been attributed to the greater dynamic range of RT-PCR.

**Table 2:** Selected genes from microarray analysis, designed pair of primers for them and its validation by quantitative RT-PCR.

Gen ID	Forward 5'→3' Reverse 5'→3'	Zscore	RQ
Rv3551	CTTGAATCTCGGTGACAGCC ACCGATTTGACCAGTTCCTC	2.473	2.780 ± 0.587
Rv3903c	AGGAGATGCTGACTGGGAT CTAGCGCCTCATTAGGGTT	3.549	18.580 ± 4.221
Rv0584	CACCCACTACGCCAATTTCT AGCGGTACCACACTGTCTC	2.824	11.890 ± 0.572
Rv0849	GAGTCCTCGTCGGAAATCTG GAACCCGAGGTGAATGTGTC	3.461	1.949 ± 0.261
Rv0176	GTTTGTGGGTTGGCTGTG CGGTTTGCCATTCATTGACG	2.967	2.250 ± 0.3
Rv1066	GCTAGTGATCGAGCGCAA CAACCCCAAGTCCAGCA	2.979	6.557 ± 0.958
Rv2780	TCCACACTCGCTACTCATCG TATCCACCAGTACCGCACCT	−3.285	−1.950 ± 0.296
Rv2895c	CTTTGAGGTTGTCGCTACCC CGACGAACACCAGCTTGAT	−4.931	−2.740 ± 0.180
Rv0954	ACTCGGCGTATTTCTGATGG CTGTCCATACGGGTCGAACT	−3.311	−2.207 ± 0.729
Rv3692	GAGCGTCAAGTCAGTGTGGA CAACTGATAGGTGCCCTCGT	−4.234	−3.556 ± 1.176
RvDB_6066	CAAGGCTAAAACTCAAAGGA GGACTTAACCCAACATCTCA	--	--

Note: Data represent mean ± SE (*n* = 3).

The up- and down-regulated genes observed in microarrays were subjected to bioinformatics analysis using the DAVID software. The results showed that two pathways converged in one up-regulated gene (Rv3551). These two pathways were geraniol degradation and 1- and 2-methyl-naphthalene degradation. The alpha subunit of coenzyme A transferase of *M. tuberculosis* (CoAt-Mt, Rv3551) is part of the unique enzyme that shares these two pathways. In the geraniol degradation, the CoAt-Mt has the function of converting geranic acid to *trans*-geranyl-CoA. In 1- and 2-methyl-naphthalene degradation this enzyme catalyzes two reactions, one of them is the conversion of naphthyl-2-methylsuccinic acid to naphthyl-2-methylsuccinyl-CoA and the other is the conversion of 2-naphthoyl-CoA to 2-naphthoate [12,13]. These pathways result interesting, because both are located only in bacteria, but not in host [14], and the alteration of these two metabolic pathways could lead to death of *M. tuberculosis*.

Geraniol, nerol, and citronellol are biosynthesized in bacteria [15]. These monoterpenes and their degradation products have been demonstrated to possess toxic effect on fungi [16]. It has been reported that geraniol displayed antifungal activity on *Saccharomyces* and *Candida.* It seems that geraniol alters the fungal membrane causing the loss of cellular potassium. This potassium leakage promotes disruption of cell processes, resulting in yeast death [17,18]. Other terpenes such as linalyl acetate, (+) menthol, thymol and farnesol showed antibacterial activity on *Staphylococcus aureus* and *Escherichia coli* [19]. Some studies showed that farnesol exerts its effect on *Staphylococcus aureus* by destabilizing the bacterial membrane [20,21]. Other studies have shown that terpenes and aromatic hydrocarbons accumulate in the membrane causing loss of its integrity [22,23,24] and decrease the regulation of potassium transport in the membrane [22]. It is likely that MDGA attached to CoAt-Mt, preventing the degradation of geraniol in *M. tuberculosis*. Thus geraniol exceeded its ordinary levels inside the cell, reaching toxic levels and destabilizing the membrane of *M. tuberculosis* producing its death.

Overexpression of one component of high-affinity ATP-driven potassium transport system (gen kdpB) is related to the leakage of potassium which is originated by injury to the membrane. As an attempt by *M. tuberculosis* to maintain an ordinary level of potassium, the gen kdpB could have been overexpressed. It is worth mentioning that the high proportion of down and overexpressed genes corresponded to functional category “cell wall and cell processes” this could be explained because this category includes genes that expresses membrane proteins, which is the place where terpenes carry out its toxic effect.

It was reported that 1- and 2-methylnaphthalene and their degradation products are toxic to 1- and 2-methylnaphthalene-degrading bacteria, such as cyanobacteria [25]. Furthermore, 1- and 2-methylnaphthalene are toxic to some fungi [26]. Thus, the inhibition of degradation of both 1- and 2-methylnaphthalene in *M. tuberculosis* could lead to accumulation of these molecules, eventually reaching toxic levels. It is important to point out that MDGA possesses neuroprotective [27], hepatoprotective [27,28], and antioxidant effects [28,29]. These properties make MDGA a potential prototype for the development of new anti-TB drugs.

### 2.3. Molecular Docking Studies

**Figure 3:**
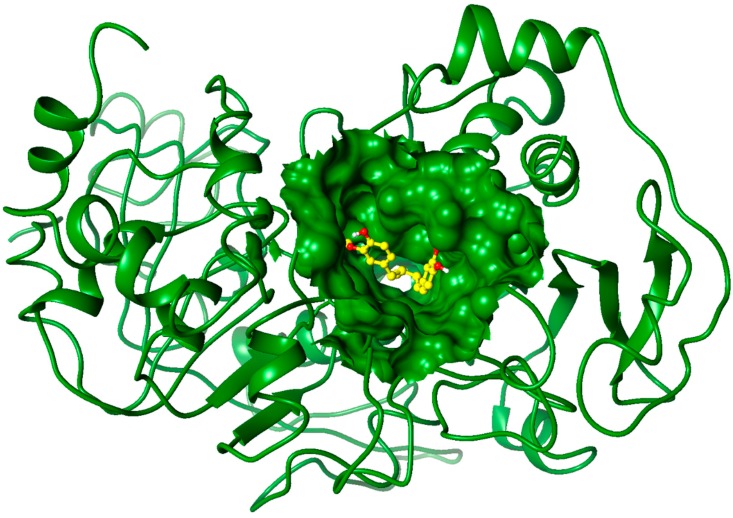
Predicted pose of the MDGA molecule on the CoAt-Mt binding site by the blind docking protocol. This figure was created with the Chimera program [34].

**Figure 4:**
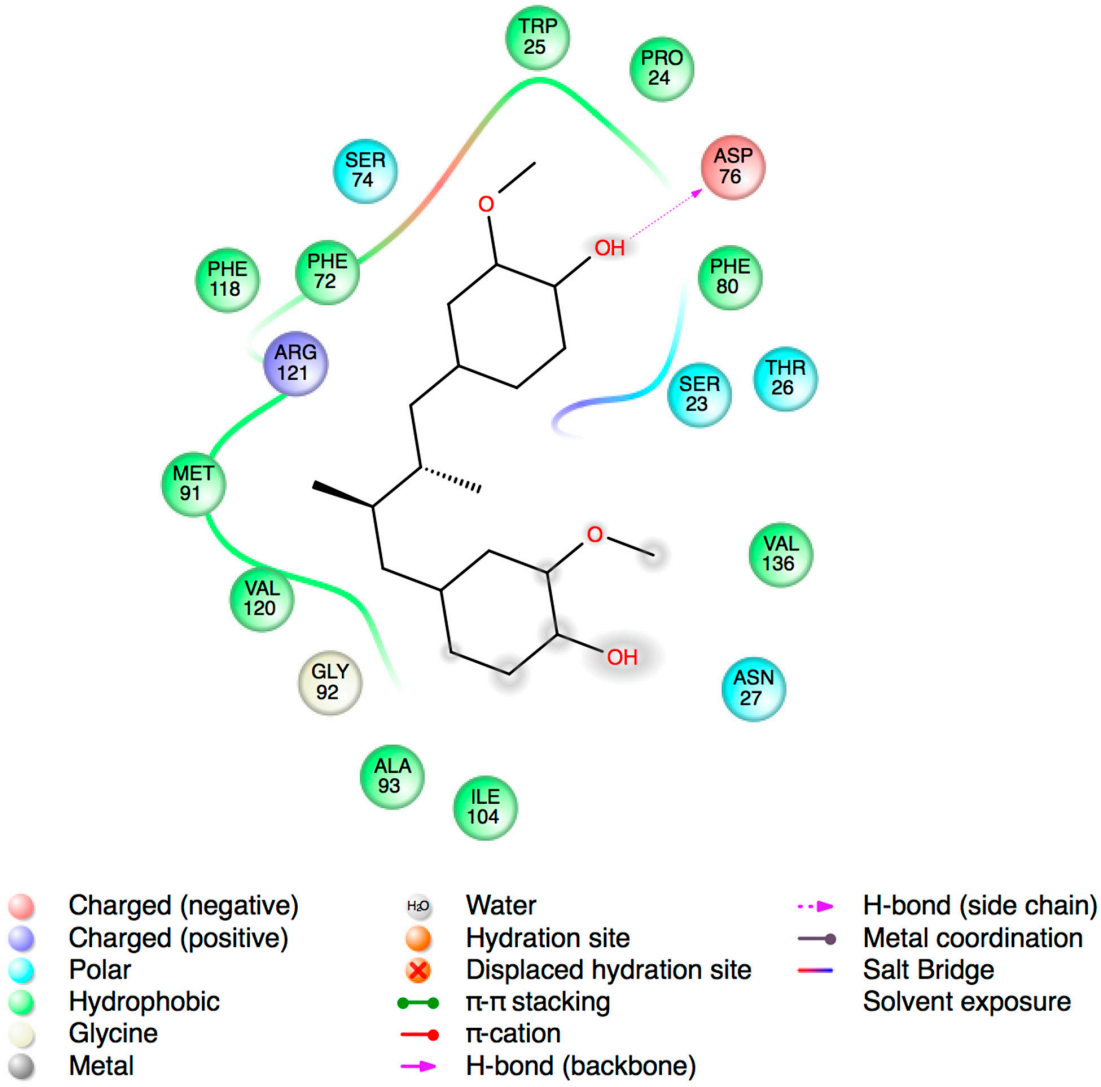
Predicted interactions of the MDGA molecule on the CoAt-Mt binding site by docking studies. The segmented lines represent hydrogen bond and its distance and the residues with red lines means interactions by Van der Waals contacts. This figure was created with the Free Maestro program [35].

To evaluate the possible binding mode of MDGA on CoAt-Mt we built a 3D model by using the protein structures of glutaconate CoA-transferase from *Acidaminococcus fermentans* [30], and acetate CoA-transferase from *E. coli* as templates [31]. In order to identify the potential binding site for the MDGA a blind docking protocol was performed using the CoAt-Mt model. Several authors have used the Autodock program to identify putative binding sites in proteins of interest [32,33]. The blind docking results showed that the MDGA molecule binds to a cavity of the CoAt-Mt that belongs to its active site (Figure 3). Thus, this binding site was selected for a focused and intensive docking calculation. The best pose according to the Autodock function scoring (−7.5 kcal/mol) was selected for analyzing its interactions with residues of the CoAt-Mt binding site. The results showed that MDGA interacts with the residues Trp25, Phe72, and Asp76 from alpha subunit (a), and Pro24, Thr26, Asn27, Met91, Gly92, Ile104, Phe118, Val120 and Arg121 from beta subunit (b), respectively. For example, the interactions of Asp76a and Asn27b residues with the MDGA molecule are mediated by hydrogen bonds, and the other ones by van der Waals contacts (Figure 4). Structural studies have suggested that residues in equivalent positions of Trp25a, Phe72a, and Asp76a are involved in the substrate binding [30,31]. Thus, the docking analysis results suggest that the mechanism of action of MDGA might be by blocking the interaction of CoAt-Mt with its substrate.

## 3. Experimental

### 3.1. Growth Curve of M. tuberculosis H37Rv with Different Concentrations of MDGA

*M. tuberculosis* H37Rv (ATCC 27294) sensitive to isoniazid, rifampicin, pyrazinamide and ethambutol was purchased from the American Type Culture Collection (ATCC, Manassas, VA, USA). *M. tuberculosis* H37Rv was grown in a conical flask (100 mL) containing Middlebrook 7H9 medium supplemented with 10% (v/v) Oleic-Albumin-Dextrose-Catalase (BBL™, MGIT™ OADC Enrichment), 0.05% (v/v) Tween 80, 0.2% (v/v) glycerol, and incubated to 37 °C for 15 days until exponential-phase was achieved. Optical density (OD) of culture was adjusted at 0.5 (λ max of 600 nm) measured in a spectrophotometer (DU 800, Beckman Coulter, San Diego, CA, USA). A stock solution of MDGA was made up in dimethyl sulfoxide (DMSO) with a concentration of 2 mg/mL. Then, 1 mL of adjusted culture was placed in each of several tubes and exposed to different concentrations of MDGA ranging from 0 to 100 µg/mL. The lignan MDGA was isolated and characterized from *L. tridentata* by our research group [6,7]. Untreated controls were prepared with adjusted cultured and DMSO (<0.05 v/v). The OD was measured in each tube from time zero, and then every 24 h to complete 120 h. The experiment was done twice on different days, and each concentration was evaluated twice in each experiment.

### 3.2. Isolation of RNA

Using the same procedure as described above, two conical flasks with 100 mL of culture of *M. tuberculosis* H37Rv with a 0.5 OD were prepared. One culture was exposed to 50 µg/mL of MDGA, and the other was exposed to DMSO (<0.05%), both cultures were incubated for 24 h. After the incubation time, each bacterial suspension was centrifuged and bacterial pellet was collected separately. RNA isolation was carried out to each bacterial pellet using the RiboPure^TM^ Bacteria kit (Cat. Num. AM1925, Invitrogen, Life Technologies, Grand Island, NY, USA), following recommendations of manufacturer. Furthermore, a treatment with DNase I was made. The quality of RNA was determined using a 1% agarose gel and the total RNA was quantified in an Eppendorf 6131 biophotometer (Eppendorf, Hauppauge, NY, USA).

### 3.3. Synthesis and Labeling of Modified cDNA

RNA quantity was adjusted at 12 µg for both treated and untreated controls. We synthesized the modified cDNA (cDNA possessing the nucleotide aminoallyluridine) using M-MLV enzyme and random decamers and the rest of reagents contained in the amino-allyl cDNA labeling kit (Cat. Num. AM1705, Invitrogen, Life Technologies, Grand Island, NY, USA). Subsequently, the remaining template RNA was hydrolyzed with 1N NaOH. The fluorescent dyes Cy3 and Cy5 (Cat. Num. RPN5661, GE Healthcare Life Sciences, Buckinghamshire Aylesbury, UK) were used for labeling modified cDNA. Each vial of fluorescent dye was dissolved with 3 µL DMSO and mixed with one specific cDNA. A NucAway Column was used to eliminate excess free color. The labeled cDNA were stored at −20 °C, until the hybridization was performed.

### 3.4. Microarray Assay

DNA chip of *M. tuberculosis* H37Rv that included 11,353 probes surveying 3,975 genes (96.69% of all genes), and designed by the MYcroarray Company (Huntsville, AL, USA) was used. The labeled cDNA of treated and untreated controls were deposited on this chip for a subsequent hybridization step at 42 °C for 24 h. The chip was supported inside a microarray hybridization chamber (Cat. Num. G2534A, Agilent Technologies, Santa Clara, CA, USA). The chip was washed three times with SSPE 1× and once with SSPE 0.25× and then dried by centrifugation. The chip was read in a GenPix 4400A Microarray Scanner (Agilent Technologies, Santa Clara, CA, USA), using wavelengths of 555 and 647 nm, to read Cy3 and Cy5, respectively. After, chip reading statistical analysis was performed using the genArise package in order to obtain Zscore values. The genes that showed a negative Zscore value means they had down-regulated expression, while genes that possessed a positive Zscore were up-regulated as a result of treatment with MDGA.

Genes with Zscore ≥ +1.5 and Zscore ≤ −1.5 were selected to carry out gene expression analysis. Both lists of genes were analyzed separately using bioinformatics tools such as Database for Annotation, Visualization and Integrated Discovery (DAVID) version 6.7 [12,13]. *M. tuberculosis* H37Rv Database (Tuberculist version 2.6) [36]; Kyoto Encyclopedia of Genes and Genomes (KEGG), [37]; National Center for Biotechnology Information (NCBI) [38]; and TB Databases (TBDB) [39].

### 3.5. Real-Time Reverse Transcription Polymerase Chain Reaction (RT-PCR)

To validate the gene expression obtained from the microarray assay, six overexpressed genes, four downexpressed genes and one housekeeping gene, were selected and employed in RT-PCR. One pair of primers was designed for each selected gene using the available Primer3 (v. 0.4.0) and Amplifx 1.5.4 softwares. RNA was obtained from treated and untreated control as previously described. Then RNAs were transcripted to cDNA using a SuperScript^®^ VILO™ cDNA Synthesis Kit (Cat. No. 11754050, Invitrogen, Life Technologies, Grand Island, NY, USA) following the manufacturer’s recommended procedure. Primers were assessed by endpoint polymerase chain reaction (PCR), before starting RT-PCR in order to ensure specific amplification of each gene. RT-PCR was performed using SYBR^®^ GreenER™ PCR SuperMix Universal (Cat. No. 11780200, Invitrogen, Life Technologies, Grand Island, NY, USA) and the comparative C_T_ method. Amplification conditions were established as follow: 50 °C, 2 min and 95 °C, 2 min as activation enzyme step; 40 amplification cycles at 95 °C, 15 s; 57 °C, 30 s and 60 °C, 1 min.

### 3.6. Sequence Retrieving, Analysis, and Homology Modeling

The amino acid sequences with accession numbers NP_218068 and NP_218069 were retrieved from the National Center for Biotechnology Information (NCBI) protein database. These sequences corresponded to the alpha and beta subunits of the CoA-transferase protein of *M. tuberculosis*, respectively. The HHPred server was used to find homologous proteins with known 3-D structures located in the Protein Data Bank (PDB). The server suggests that the structures of the glutaconate CoA-transferase from *Acidaminococcus fermentans* (PDB ID: 1K6D) and acetate CoA-transferase from *Escherichia coli* (PDB ID: 1POI) were the best templates for the homology modeling protocol. The 3D model of the CoA-transferase of *M. tuberculosis* was built based on templates with the MODELLER 9v11 program [40]. The quality of the homology model was verified using the ANOLEA program [41].

### 3.7. Molecular Docking

The 3D structure of the MDGA was retrieved from the PubChem database [42]. The hydrogens and charges on receptor and ligand were assigned with the Chimera program [34]. The pdbqt files for docking simulations were generated using the AutoDock Tools interface [43]. The docking of MDGA acid molecule on CoAt-Mt model was performed with Autodock 4.2 program [44]. In order to identify potential binding sites of MDGA on CoAt-Mt, a blind docking procedure was performed. Firstly, the whole surface of protein receptor was defined as search spaces. The grid size in each dimension (x, y, and z) was 126 Å, with its center at the middle of the protein. The other default optimization parameters were maintained for docking simulation, except for the number of GA runs, population size and maximum number of evaluations, which were set to 100, 250 and 25,000,000, respectively. The predicted binding site for the MDGA was used for a second round of docking simulations with smaller search spaces (grids) around of predicted binding site. These grid sizes in each dimension (x, y, and z) were 60 Å, grid points separated by 0.375 Å, with its center in 33, 4, and −14 for x, y and z, respectively. The analysis of Autodock predicted poses was performed with the AutoDock Tools interface and Chimera program. 

## 4. Conclusions

We conclude that the overexpression of CoAt-Mt in both microarray and RT-PCR assays and the resulting stable interactions of MDGA with the active site of CoAt-Mt in molecular docking, provide strong evidence of inhibition of this enzyme present in both the geraniol and 1- and 2-methylnaphthalene degradation pathways, therefore, CoAt-Mt is a potential drug target in *M. tuberculosis*.

## Supplementary Materials

Supplementary materials can be accessed at: http://www.mdpi.com/1420-3049/19/12/20170/s1.
