# Supplementary material for: Potential Mechanism of Action of meso-Dihydroguaiaretic Acid on Mycobacterium tuberculosis H37Rv
Source: Molecules. 2014 Dec 2;19(12):20170–82. doi: 10.3390/molecules191220170 (PMC6271217; doi:10.3390/molecules191220170)

Supplementary Materials

Figure S1. Lignan *meso*-dihydroguaiaretic acid with antimycobacterial activity.

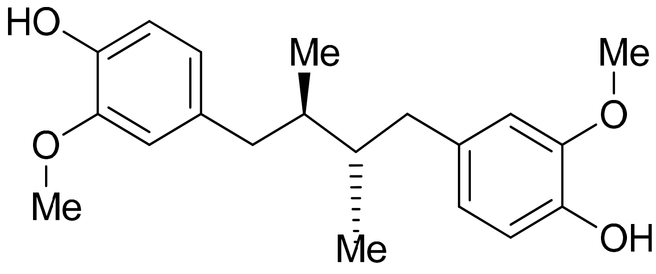

Figure S2. Predicted interactions of the MDGA molecule on the CoAt-Mt binding site by docking studies.The segmented lines represent hydrogen bond and its distance and the residues with red lines means interactions by Van der Waals contacts.

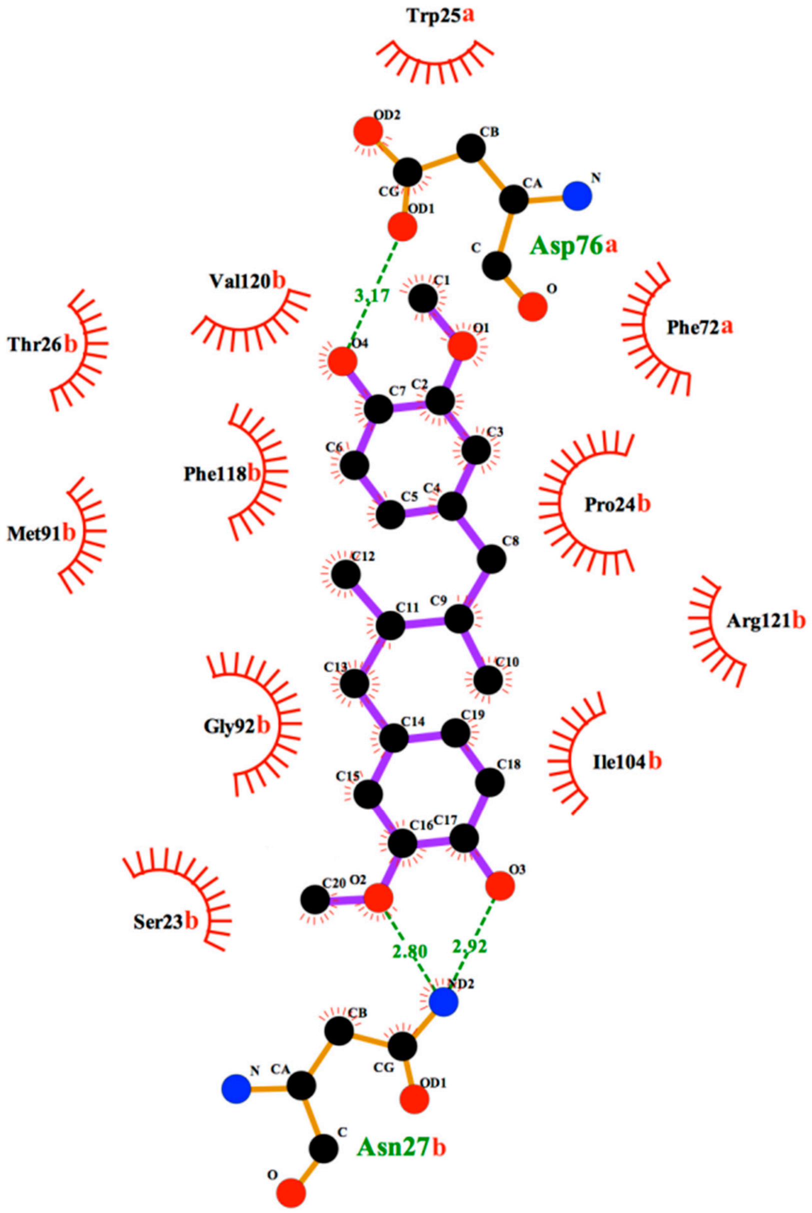

Supplement: Supplementary file 1 [file molecules-19-20170-s001.pdf]
